# Graft preservation confers myocardial protection during coronary artery bypass grafting

**DOI:** 10.3389/fcvm.2022.922357

**Published:** 2022-07-28

**Authors:** Philipp Szalkiewicz, Maximilian Y. Emmert, Paul P. Heinisch, Zsuzsanna Arnold, Ingo Crailsheim, Markus Mach, Thomas Aschacher, Martin Grabenwöger, Bernhard Winkler

**Affiliations:** ^1^Karl Landsteiner Institute for Cardiac and Vascular Surgical Research, Vienna, Austria; ^2^Deutsches Herzzentrum Berlin, Berlin, Germany; ^3^Clinic for Cardiovascular Surgery, Charité University Medicine Berlin, Berlin, Germany; ^4^German Heart Centre Munich, Technical University Munich, Munich, Germany; ^5^Vienna Health Association, Vienna, Austria; ^6^Medical University of Vienna, Vienna, Austria; ^7^Clinical Department of Cardiac Surgery, University Department of Surgery, Medical University of Vienna, Vienna, Austria; ^8^Sigmund Freud University Vienna, Vienna, Austria

**Keywords:** vein graft preservation, storage solutions, myocardium, protection, coronary artery

## Abstract

**Background:**

During on-pump coronary artery bypass grafting (ONCAB), graft flushing for distal anastomoses testing also perfuses the downstream myocardium. This single-center retrospective study evaluated the impact of specific preservation solutions on myocardial protection during ONCAB.

**Materials and methods:**

Between July 2019 and March 2020 either DuraGraft (DG) or 0.9% Saline/Biseko (SB) was applied to 272 ONCAB. Overall, 166 patients were propensity-matched into two groups. Cardiac enzymes [high-sensitive Troponin I (hs-TnI) and creatine kinase (CK)] were evaluated 7 days post-surgery.

**Results:**

Post-surgery, hs-TnI values were significantly lower from 3 to 6 h (h) up to 4 days in the DG group: 3–6 h: 4,034 ng/L [IQR 1,853–8,654] vs. 5,532 ng/L [IQR 3,633—8,862], *p* = 0.05; 12–24 h: 2,420 ng/L [IQR 1,408–5,782] vs. 4,166 [IQR 2,052–8,624], *p* < 0.01; 2 days: 1,095 ng/L [IQR 479–2,311] vs. 1,564 ng/L [IQR 659–5,057], *p* = 0.02 and at 4 days: 488 ng/L [IQR 232–1,061] vs. 745 ng/L [IQR 319–1,820], *p* = 0.03. The maximum value: 4,151 ng/L [IQR 2,056–8,621] vs. 6,349 ng/L [IQR 4,061–12,664], *p* < 0.01 and the median area under the curve (AUC): 6,146 ng/L/24 h [IQR 3,121–13,248] vs. 10,735 ng/L/24 h [IQR 4,859–21,484], *p* = 0.02 were lower in the DG group. CK values were not significantly different between groups: maximum value 690 [IQR 417–947] vs. 631 [464–979], *p* = 0.61 and AUC 1,986 [1,226–2,899] vs. 2,081 [1,311–3,063], *p* = 0.37.

**Conclusion:**

Repeated graft flushing with DG resulted in lower Troponin values post-surgery suggesting enhanced myocardial protection compared to SB. Additional studies are warranted to further assess the myocardial protection properties of DG.

## Introduction

Although graft storage solutions (GSS) are known to influence graft failure rates following coronary artery bypass grafting (CABG), their impact on myocardial protection in the context of ischemia reperfusion injury (IRI) during cardiopulmonary bypass remains unknown. Saphenous vein grafts (SVG) remain the most commonly used conduits in CABG procedures (1). However, SVGs are prone to vein graft disease and failure. While graft failure and the documented early pathohistological or functional changes in SVG are initiated by various means including underlying harvesting techniques (2), inadequate tissue protection during graft storage appears to be the main trigger (3). Although saline and autologous blood are frequently used as GSS, their use is inadequate to maintain graft patency compared to subsequent GSS (3–5). DuraGraft (DG; Marizyme, Jupiter FL, United States) is an endothelial damage inhibitor, formulated into a preventive solution to protect the integrity and function of the graft when used in this manner. Several *in vitro* studies have shown the protective potential of DG (6–9). Recent research demonstrated reduced graft intimal hyperplasia in SVGs treated with DG (10), and a large-scale retrospective analysis from the Boston West Roxbury Veterans Hospital suggested that treatment of SVGs is associated with significantly lower rates of repeat revascularization and major adverse cardiac events compared to saline (11). While DG has demonstrated its capacity for vascular graft preservation, its potential for myocardial protection in on-pump coronary artery bypass grafting (ONCAB) after intracoronary perfusion by graft flushing for distal anastomosis leak testing during cardiopulmonary bypass remains unclear. In this study, we examined the impact of DG on myocardial protection. This was evaluated by assessing the post-operative biomarker release profile following SVG storage in DG and its downstream intracoronary infusion (*via* the distal anastomosis) in patients undergoing ONCAB procedures and compared to 0.9% Saline/Biseko (SB; control).

## Patients and methods

### Study design

This study was a retrospective single-center analysis of cardiac biomarker release in patients undergoing ONCAB whose SVGs were treated with DG or SB at the Vienna Heart Center, Floridsdorf Nord, Austria between July 2019 and March 2020. Both GSS were applied separately in different subsequent time intervals. Every patient underwent ONCAB with the treatment of at least one SVG with one of the indicated GSS. Follow-up was conducted by outpatient management and telephone follow-up to obtain information on post-operative myocardial infarction (MI), repeat revascularization, and death. Mortality data were obtained by request from the mortality registry of Statistic Austria, the Austrian statistical office. Ethics approval for the study protocol was received by the local ethics committee.

### Surgical technique

In all ONCAB procedures, aortic cross-clamping was implemented. Cardioplegia was applied in standard antegrade and/or retrograde fashion using cold blood cardioplegia. SVGs were harvested by either the open or endoscopic technique. After harvesting and preparation, grafts were stored and flushed either in DG or SB until distal anastomosis was carried out. If arterial grafts were used, only the radial artery was stored in either GSS. After completion of the distal anastomosis in sequential, graft-to-graft, or direct single anastomosis fashion, a careful repeated graft flushing with the respective GSS was performed for leak testing.

### Laboratory evaluation of cardiac markers

Cardiac enzymes high-sensitive Troponin I (hs-TnI) and creatine kinase (CK) were measured pre-surgery, 1–3, 3–6, and 12–24 h after CABG, and once daily up to 7 days post-CABG. Measurement of both cardiac markers was conducted each by the Abbott Alinity assay. Hs-TnI-related detection limits are 1 ng/L, with sex-specific normal upper reference limit of 34.2 ng/L for men and 15.6 ng/L for women. CK-related detection limits are 7 U/L, with sex-specific normal upper reference limit of 200 U/L for men and 168 U/L for women.

### Graft storage solutions

DuraGraft (Marizyme, Jupiter FL, United States) is a GSS which preserves endothelial function and structure during CABG procedures. DG contains L-glutathione, L-ascorbic acid, L-arginine, glucose, and balanced salts for minimizing ischemic and metabolic damage to the conduits during graft preservation, graft handling, and IRI. Moreover, the pH is buffered in the physiologic range. Biseko (Biotest Pharma GmbH, Dreieich, Germany), which is known to be used off-label for graft storage, is an ionized plasma derivate containing human serum protein, albumin, and human immunoglobulin. Saline 0.9% was added due to the lower volume of Biseko. Additionally, heparin was added to both DG and SB during their preparation for surgical application.

### Statistical analysis

**FIGURE 1:**
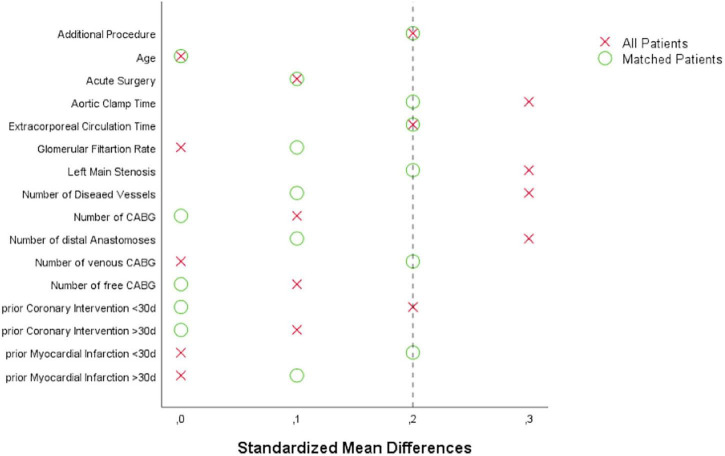
Love plot visualizing covariate balancing according to standardized mean differences before and after propensity score matching: CABG, coronary artery bypass grafting.

Categorical variables, shown as numbers and percentage, were compared by Chi-Square test or Fisher-Exact test for small sample size correction. Continuous variables, expressed as median and interquartile range (IQR), were compared by Mann–Whitney–*U*-test. Propensity score matching (PSM) was performed to minimize potentially responsible bias for the evaluation of biomarker values and cardiac adverse events between both study groups. Matching was conducted by a multivariate logistic model, which consisted of 1-to-1 patient pair formation, nearest-neighbor matching, and no case-replacement. The corresponding caliper of 0.15 was chosen. The model incorporated the following variables: additional cardiac procedure, age, acute surgery, aortic clamp time, extracorporeal circulation time, glomerular filtration rate, left main stenosis, number of diseased vessels, number of CABG, number of distal anastomoses, number of venous CABG, number of free CABG, prior percutaneous coronary intervention (PCI) ≤30 days, prior PCI >30 days, prior MI ≤30 days, and prior MI >30 days. Covariate balancing was evaluated according to calculating standardized mean differences with a value of ≤0.2 considered as sufficient equilibrium of these covariates between the groups (Figure 1). Differences in prevalence of baseline and procedural variables between both groups of patients were evaluated by diagnostic odds ratios according to logistic regression and effect size estimation according to standardized mean differences. The area under the curve (AUC) was calculated from the plasma biomarker concentrations vs. the time after surgery. Data analysis was conducted by SPSS statistical software version 25 (IBM Corp, Armonk, NY, United States). *P*-values of <0.05 were considered statistically significant.

## Results

### Pre- and peri-procedural data

**FIGURE 2:**
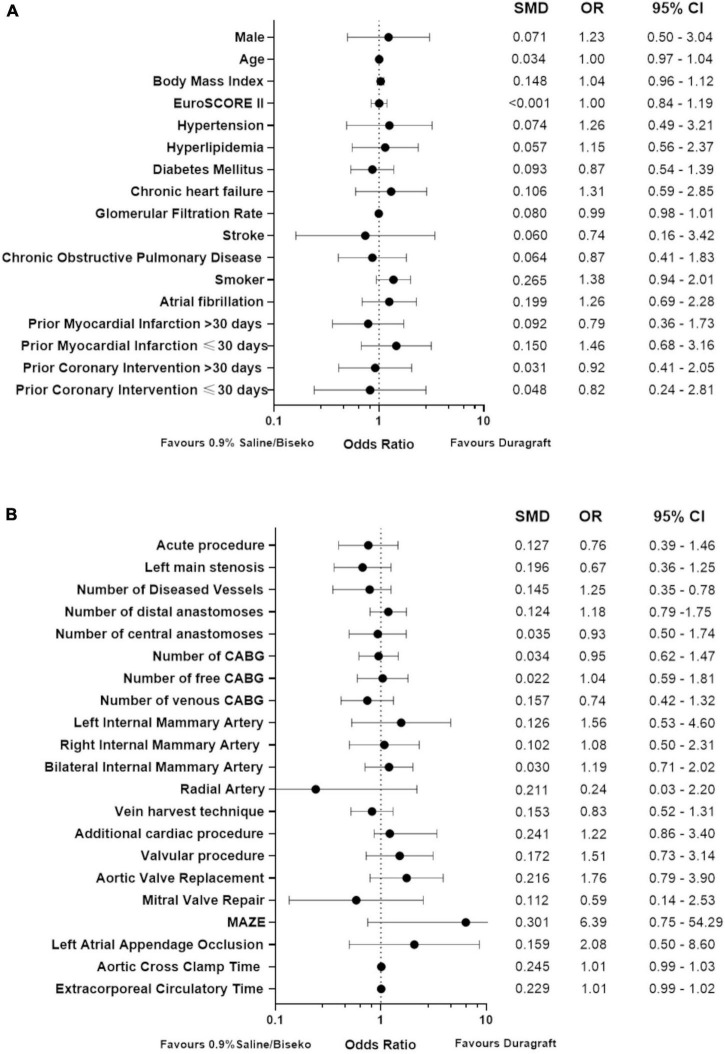
Forest plot presenting diagnostic odds ratios and effect size of baseline **(A)** and procedural variables **(B)** after propensity score matching; CABG, coronary artery bypass grafting; SMD, standardized mean differences, OR, odds ratio, 95% CI, 95% confidence interval.

The SVGs of 100 patients were treated with DG and 172 patients with SB. After PSM, 83 patients within each group were selected. The corresponding Love plot (Figure 1) provides visualization of covariate balancing of all included baseline (Table 1) and procedural variables (Table 2) in the propensity model. The included covariates were equally distributed between both groups according to standardized mean differences, which did not exceed rounded maximum values of 0.2. Therefore, the conducted PSM might be considered valid. Moreover, diagnostic odds ratios with associated 95% confidence intervals and effect size according to given standardized mean differences of all baseline and procedural variables of the propensity-matched patient population are presented in a given Forest plot (Figure 2). After PSM, only documented smokers (DG: 17.9 vs. SB: 30.0%; *p* = 0.03) and those on whom the concomitant MAZE procedure (DG: 7.2 vs. SB: 1.2%; *p* = 0.12) were conducted were distributed unequally between both groups according to an increased effect size for the given variables, with values of standardized mean differences of 0.265; OR: 1.38; 95% CI: 0.94–2.01, and 0.301; OR: 6.39; 95% CI: 0.75–54.29, respectively. Although both variables revealed a higher prevalence within the DG group, both groups appeared overall homogenized after PSM as the remaining baseline and procedural characteristics were equally distributed within the patient population (Figure 2).

**TABLE 1:** Patient baseline characteristics.

	Unmatched			Matched		
		
*n* (%)	DuraGraft *n* = 100	Saline/Biseko *n* = 172	*p*-value	DuraGraft *n* = 83	Saline/Biseko *n* = 83	*p*-value
Male	89 (89.0)	136 (79.1)	0.04	73 (88.0)	71 (85.5)	0.65
Age (years), median (IQR)	72(62−75)	70(61−76)	0.77	71(62−75)	69(62−75)	0.81
BMI, median (IQR)	27.7(24.9−30.4)	27.2(24.8−30.3)	0.52	28.4(25.2−30.8)	26.9(24.9−30)	0.22
EuroSCORE II, median (IQR)	1.7(1.1−3.3)	1.7(1.1−3.1)	0.59	1.6(0.9−3.1)	1.6(0.9−3.1)	0.81
Hypertension	87 (87.0)	147 (85.5)	0.73	72 (86.7)	74 (89.2)	0.63
Hyperlipidemia	78 (78.0)	135 (78.5)	0.93	65 (78.3)	63 (75.9)	0.71
Diabetes mellitus			0.89			0.82
IDDM	7 (7.0)	13 (7.6)		6 (7.2)	8 (9.6)	
NIDDM	37 (37.0)	68 (39.5)		30 (36.1)	31 (37.3)	
None	56 (56.0)	91 (52.9)		47 (56.6)	44 (53)	
Chronic heart failure	24 (26.1)	32 (19.8)	0.24	18 (22.2)	14 (17.9)	0.50
GFR (ml/min), median (IQR)	76.5(57−88)	73(59−86)	0.66	78(59−90)	74(64−88)	0.92
Stroke	4 (4.0)	17 (9.9)	0.80	3 (3.6)	4 (4.8)	1.00
COPD	23 (27.4)	49 (35.8)	0.20	18 (26.1)	20 (29)	0.70
Smoker			0.001			0.03
Active	19 (20.4)	54 (32.0)		14 (17.9)	24 (30.0)	
Former	31 (33.3)	23 (13.6)		27 (34.6)	14 (17.5)	
None	43 (46.2)	92 (54.4)		37 (47.4)	42 (52.5)	
Atrial fibrillation	15 (15.0)	28 (16.3)	0.78	13 (15.7)	11 (13.3)	0.66
Prior MI > 30 days	18 (18.2)	28 (16.3)	0.69	14 (16.9)	17 (20.5)	0.55
Prior MI ≤ 30 days	21 (21.0)	36 (21.1)	0.99	19 (22.9)	14 (16.9)	0.44
Prior PCI > 30 days	15 (15.0)	29 (16.9)	0.69	14 (16.9)	15 (18.1)	0.84
Prior PCI ≤ 30 days	5 (5.0)	16 (9.3)	0.20	5 (6.0)	6 (7.2)	0.76

BMI, body mass index; COPD, chronic obstructive pulmonary disease; EuroSCORE II, updated European system for cardiac operative risk evaluation; GFR, glomerular filtration rate; IDDM, insulin dependent diabetes mellitus; IQR, interquartile range; NIDDM, not insulin dependent diabetes mellitus; Prior MI ≤ 30 days/ > 30 days, preoperative myocardial infarction within/beyond 30 days prior surgery; Prior PCI ≤ 30 days/ > 30 days, preoperative percutaneous coronary intervention within/beyond 30 days prior surgery.

**TABLE 2:** Procedural characteristics and concomitant procedures.

	Unmatched			Matched		
		
*n* (%)	DuraGraft	Saline/Biseko	*p*-value	DuraGraft	Saline/Biseko	*p*-value
	*n* = 100	*n* = 172		*n* = 83	*n* = 83	
Acute procedure	29 (29.0)	59 (34.3)	0.37	25 (30.1)	30 (36.1)	0.41
Left main stenosis	31 (31.0)	77 (44.8)	0.03	30 (36.1)	38 (45.8)	0.21
Number of vessels diseased, median (IQR)	3(3−3)	3(2−3)	0.18	3(2−3)	3(2−3)	0.44
Number distal anastomoses, median (IQR)	3(3−3)	3(2−3)	0.02	3(3−3)	3(2−3)	0.36
Number of central anastomoses, median (IQR)	1(1−2)	1(1−2)	0.91	1(1−2)	1(1−2)	0.95
Number of CABG, median (IQR)	3(2−3)	3(2−3)	0.54	3(2−3)	3(2−3)	0.72
Number of free CABG, median (IQR)	2(1−2)	2(1−2)	0.69	2(1−2)	2(1−2)	0.83
Number of venous CABG, median (IQR)	1(1−2)	1(1−2)	0.77	1(1−2)	1(1−2)	0.41
LIMA			0.34			0.43
*In situ*	93 (93.0)	151 (87.8)		77 (92.8)	73 (88)	
Free graft	0 (0.0)	2 (1.2)		0 (0.0)	1 (1.2)	
None	7 (7.0)	19 (11.0)		6 (7.2)	9 (10.8)	
RIMA			0.60			0.41
*In situ*	8 (8.0)	9 (5.2)		8 (9.6)	4 (4.8)	
Free graft	9 (9.0)	19 (11.0)		9 (10.8)	12 (14.5)	
None	83 (30.5)	144 (83.7)		66 (79.5)	67 (80.7)	
BIMA	17 (17.0)	28 (16.3)	0.88	17 (20.5)	16 (19.3)	0.85
Radial artery	1 (1.0)	7 (4.1)	0.27	1 (1.2)	4 (4.8)	0.37
Vein harvest			0.94			0.67
Endoscopic	41 (54.7)	62 (54.9)		35 (56.5)	27 (50.9)	
Surgical	20 (26.7)	28 (24.8)		16 (25.8)	13 (24.5)	
Both	14 (18.7)	23 (20.4)		11 (17.7)	13 (24.5)	
**Concomitant surgeries**						
Additional cardiac procedure	39 (39)	51 (29.8)	0.12	28 (33.7)	19 (22.9)	0.12
Valvular procedure	32 (32)	42 (24.4)	0.18	22 (26.5)	16 (19.4)	0.36
Aortic valve replacement	14 (16.3)	31 (18)	0.17	19 (22.9)	12 (14.5)	0.16
Mitral valve replacement	4 (4.0)	3 (1.7)	0.27	0 (0.0)	0 (0.0)	
Mitral valve repair	3 (3.0)	12 (7)	0.17	3 (3.6)	5 (6)	0.72
Tricuspid valve replacement	0 (0.0)	0 (0.0)		0 (0.0)	0 (0.0)	
Tricuspid valve repair	1 (1.0)	2 (12)	1.00	0 (0.0)	0 (0.0)	
MAZE	8 (8.0)	7 (4.1)	0.17	6 (7.2)	1 (1.2)	0.12
LAA	8 (8.0)	8 (4.7)	0.26	6 (7.2)	3 (3.6)	0.50
**Procedural characteristics**						
Aortic cross clamp time (minutes), median (IQR)	79(63−103)	71(57.3−91)	0.01	74(62−93)	70(57−89)	0.07
Extracorporeal circulatory time (minutes), median (IQR)	123(102−143)	112.5(97.3−139)	0.09	119(101−140)	110(93−135)	0.14

BIMA, bilateral internal mammary artery; CABG, coronary artery bypass grafting; IQR, interquartile range, LAA, left atrial appendage occlusion; LIMA, left internal mammary artery; RIMA, right internal mammary artery.

### Evaluation of cardiac biomarkers

**FIGURE 3:**
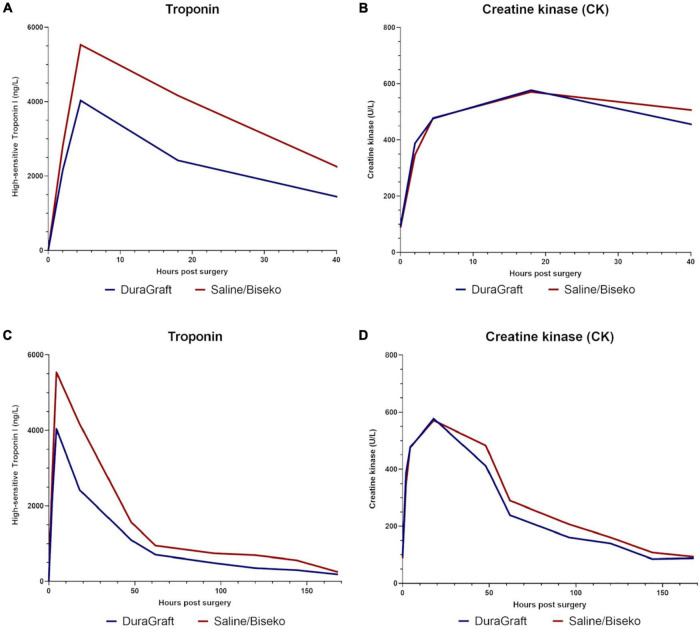
Median values of high-sensitive Troponin I in nanogram/liter and creatine kinase in units/liter within the first hours post-surgery **(A,B)**, and during the overall hospital stay **(C,D)**.

Data on post-procedural laboratory values (Tables 3, 4) revealed a typical post-operative monophasic course for both hs-TnI and CK (Figure 3). Hs-TnI values were comparable pre-surgery (DG: 11 [5–28] vs. SB: 8 [3–28], *p* = 0.22). Post-surgery, hs-TnI values were significantly lower from 3 to 6 h until the measurement at 4 days in the DG group: 3–6 h: 4,034 ng/L [IQR 1853–8654] vs. 5,532 ng/L [IQR 3633–8862], *p* = 0.05; 12–24 h: 2,420 ng/L [IQR 1408–5782] vs. 4166 [IQR 2052–8624], *p* < 0.01; 2 days: 1,095 ng/L [IQR 479–2311] vs. 1,564 ng/L [IQR 659–5057], *p* = 0.02, and at 4 days: 488 ng/L [IQR 232–1061] vs. 745 ng/L [IQR 319–1820], *p* = 0.03. Noteworthy, hs-TnI values only reached borderline significance at 3 days: 709 ng/L [IQR 321–1696] vs. 950 ng/L [IQR 332–2105], *p* = 0.08. The maximum value and the median AUC were also significantly lower after graft treatment with DG (maximum value: 4,151 ng/L [IQR 2056–8621] vs. 6,349 ng/L [IQR 4061–12664], *p* < 0.01 and AUC: 6,146 ng/L/24 h [IQR 3121–13248] vs. 10,735 ng/L/24 h [IQR 4859–21484], *p* = 0.02). CK values in the DG group appeared to be slightly higher at 1–3 h (388 U/L [IQR 286–563) vs. 347 U/L [IQR 267–483], *p* = 0.05, while lower values in DG patients at 4 days (161 U/L, [IQR 99–276] vs. 208 U/L (136–324), *p* = 0.07) and at 6 days (85 U/L [IQR 55–169] vs. 108 U/L [IQR 74–160], *p* = 0.09) did not reach complete statistical significance.

**TABLE 3:** Median values and interquartile ranges of high-sensitive Troponin I in nanograms/liter post-CABG after propensity matching.

Troponin, median (IQR), *n*	DuraGraft *n* = 83	Saline/Biseko *n* = 83	*p*-value
Pre-surgery	11 (5-28), 73	8 (3-28), 80	0.22
1-3 h	2147 (1155-3855), 80	2790 (1425-4033), 78	0.25
3-6 h	4034 (1853-8654), 63	5532 (3633-8862), 62	0.05
12-24 h	2420 (1408-5782), 83	4166 (2052-8624), 83	<0.01
2 days	1095 (479-2311), 83	1564 (659-5057), 83	0.02
3 days	709 (321-1696), 75	950 (332-2105), 79	0.08
4 days	488 (232-1061), 66	745 (319-1820), 69	0.03
5 days	354 (123-941), 56	696 (176-1318), 46	0.17
6 days	301 (149-915), 53	561 (241-1061), 58	0.18
7 days	186 (83-588), 58	249 (70-697), 44	0.81
Max value	4151 (2056-8621), 83	6349 (4061-12664), 83	<0.01
AUC	6146 (3121-13248), 83	10735 (4859-21484), 83	0.02

AUC, area under the curve; CABG, coronary artery bypass grafting; IQR, interquartile range.

**TABLE 4:** Median values and interquartile ranges of creatine kinase in units/liter post-CABG after propensity matching.

Creatine kinase, median (IQR), *n*	DuraGraft *n* = 83	Saline/Biseko *n* = 83	*p*-value
Pre-surgery	93 (71–150), 75	89 (64–120), 82	0.32
1–3 h	388 (286–563), 83	347 (267–483), 81	0.05
3–6 h	476 (303–730), 63	478 (373–639), 60	0.75
12–24 h	578 (339–815), 82	571 (400–863), 83	0.56
2 days	412 (249–654), 83	483 (308–684), 83	0.14
3 days	239 (153–396), 76	290 (160–460), 79	0.16
4 days	161 (99–276), 67	208 (136–324), 70	0.07
5 days	140 (95–280), 59	161 (95–271), 48	0.62
6 days	85 (55–169), 52	108 (74–160), 58	0.09
7 days	89 (56–158), 56	93 (66–123), 43	0.75
Max value	690 (417–947), 83	631 (464–979), 83	0.61
AUC	1986 (1226–2899), 83	2081 (1311–3063), 83	0.37

AUC, area under the curve; CABG, coronary artery bypass grafting; IQR, interquartile range.

### Adverse events

After PSM, the median hospital stay in days (DG: 16.5 [IQR 12–22] vs. SB: 15 [IQR 12–22]; *p* = 0.62), all-cause mortality (DG: 6.0 vs. SB: 2.4%, *p* = 0.44), and cardiac-related mortality (DG: 2.4 vs. SB: 0.0%, *p* = 0.70) did not differ between both groups over the median follow-up of 4 (IQR 0–22) months (Table 5). Noteworthy, all mortality events occurred within 1-year of follow-up. Progression of heart failure was the cause of death in the four patients with cardiac death.

**TABLE 5:** Cardiac adverse events after matching over the median follow-up of 4 (IQR 0–22) months.

*n* (%)	DuraGraft	Saline/Biseko	*p*-value
	*n* = 83	*n* = 83	
Mortality	5 (6.0)	2 (2.4)	0.44
30-day Mortality	3 (3.6)	2 (2.4)	1.00
Cardiac related mortality	2 (2.4)	0 (0.0)	0.70
MI	1 (1.2)	3 (3.6)	0.62
PCI	1 (1.2)	6 (7.2)	0.18
Stroke	3 (3.6)	2 (2.4)	1.00
New onset of atrial fibrillation	23 (27.7)	16 (19.3)	0.27
AV-Block (II° and III°)	1 (1.2)	1 (1.2)	1.00
Pacemaker implantation	1 (1.2)	2 (2.4)	1.00
Pneumonia	7 (8.4)	7 (8.4)	1.00
Hospital stay in days, median (IQR)	16.5 (12–22)	15 (12–22)	0.624

AV-Block, atrioventricular block; Cardiac related Death, composite endpoint of events of progressing or acute onset of heart failure; MI, myocardial infarction; PCI, percutaneous coronary intervention.

### Comment

This study shows that the use of DG for leak testing during distal anastomosis and its subsequent application to the downstream myocardium appears to be associated with improved myocardial protection in patients undergoing ONCAB, identified by significantly lower hs-TnI levels including the maximum value and AUC during the early post-operative phase after CABG when compared to SB. In contrast, values of CK were comparable between both groups within the observation period, which may be due to CK being a non-specific cardiac enzyme, while Troponin I is highly specific for myocardial injury (12).

Several factors influence cardiac marker increase, such as the complexity of the CABG procedure (13, 14) and renal impairment (15). From a clinical perspective, the observed overall lower early post-operative Troponin levels in the DG group might be a predictor of better overall patient outcomes when considering that increased Troponin values have been described to predispose to increased early and long-term mortality and cardiac-related complication rates (13, 14, 16).

In our study, peak values for hs-TnI were reached within temporal proximity of 3–6 h in both groups post-surgery, and the most significant difference in absolute hs-TnI values was seen at 12–24 h after surgery. Afterward, in both groups, values decreased in a logistic linear fashion. Overall, enzyme increase during ONCAB indicates ischemic myocardial damage (17). Sufficient administration of cardioplegia for adequate myocardial protection during on-pump cardiac surgery is crucial. Insufficient protection of the heart during on-pump runs leads to increased local metabolic stress which can be particularly problematic for the right ventricle and in the context of inefficient retrograde application of cardioplegia (18). Since DG has been designed to prevent IRI in vascular conduits, one may hypothesize that additional specific administration of DG into the downstream myocardium *via* systematic flushing of the distal anastomosis during leak testing may protect the downstream myocardium from IRI as well and enhance myocardial protection, which was reflected by significantly lower post-operative troponin values in our study.

DuraGraft, as well as its precursor GALA solution, on which the basis of DG was developed, has demonstrated superiority in preserving tissue functionality over saline and other GSS in human venous and free arterial conduits by reducing oxidative stress (6, 8, 9) and through the actions of L-arginine (a key component of DG) to sustain NO concentration, thereby maintaining endothelial function and preventing hyperplasia (19). In direct comparison, saline fails to prevent oxidative damage and downstream IRI (6), while its acidic pH (5.5) might induce solution damage as well (3).

On the other hand, Biseko enables superior preservation of graft patency by decreased risk of vasospasm (20) and endothelial pressure damage due to graft flushing (21) when compared to saline; however, Biseko does not protect against ischemic injury. One may speculate that in line with its established protective impact on the endothelium of vascular conduits (6, 8, 9), DG might also have similar beneficial effects on the endothelial integrity and function of the coronary vasculature in the treated territory, which could ultimately explain myocardial protection. This might be particularly driven by L-, which is one of the main components in DG, and which is known (i) to enhance coronary blood flow by enhanced vasodilation following intracoronary application (22) and simultaneously (ii) to reduce IRI (23).

However, if and to what extent the observed positive effects on myocardial protection and its underlying mechanisms are directly comparable to the previously described protective impact of DG on vascular conduits needs further in-depth investigation. Nevertheless, if proven valid, this concept could have great clinical relevance in future on-pump revascularization strategies, especially when considering the frequently seen challenges with inadequate myocardial protection and associated poor recovery of the right ventricle after CABG procedures.

### Limitations

This was a retrospective, non-randomized study, hence all established limitations do apply. No additional cardiac imaging for evaluation of myocardial protection and graft patency was conducted. Intraoperative intracoronary flow measurement could not be evaluated retrospectively. Measurement of the myocardial fraction of CK and CK-MB was not done in this study, but may have yielded statistically significant differences, and thus, may be considered for future studies. Finally, this study was not powered for clinical outcome events.

## Conclusion

In this study, the administration of DG into the downstream myocardium during graft flushing for leak testing of the distal anastomosis was associated with improved perioperative myocardial protection as measured by significantly lower troponin levels when compared to SB in patients undergoing ONCAB.

## Data availability statement

The original contributions presented in the study are included in the article/supplementary material, further inquiries can be directed to the corresponding author.

## Ethics statement

The studies involving human participants were reviewed and approved by the Ethic committee of the city of Vienna. The patients/participants provided their written informed consent to participate in this study.

## Author contributions

PS: conceptualization, data curation, formal analysis, investigation, methodology, project administration, supervision, validation, visualization, writing – original draft, and writing – review and editing. ME and BW: conceptualization, investigation, methodology, project administration, supervision, validation, and writing – review and editing. PH, ZA, IC, MM, TA, and MG: conceptualization and writing – review and editing. All authors contributed to the article and approved the submitted version.

## Abbreviations

AUC: area under the curve
CABG: coronary artery bypass grafting
CK: creatine kinase
DG: duragraft
GSS: graft storage solution
Hs-TnI: high-sensitive Troponin I
IRI: ischemia reperfusion Injury
IQR: interquartile range
MI: myocardial infarction
ONCAB: On-pump coronary artery bypass grafting
PCI: percutaneous coronary intervention
PSM: propensity score matching
SB: saline/biseko
SVG: saphenous vein grafts.
